# The Essential Role of Cytokinin Signaling in Root Apical Meristem Formation during Somatic Embryogenesis

**DOI:** 10.3389/fpls.2015.01196

**Published:** 2016-01-05

**Authors:** Lei Wang, Kang Chong

**Affiliations:** Key Laboratory of Plant Molecular Physiology, Institute of Botany, Chinese Academy of SciencesBeijing, China

**Keywords:** somatic embryogenesis, hypophysis, shoot-root axis, auxin gradients, cytokinin response, RAM establishment

Plant tissue culture and subsequent organogenesis have been regarded as part of the scientific roots of modern plant biotechnology (Sussex, 2008). Establishments of shoot apical meristem (SAM) and root apical meristem (RAM) are critical steps for somatic embryogenesis (Scheres, 2007). *WUSCHEL* (*WUS*), a homeodomain protein, controls stem cell differentiation and maintenance and its ectopic expression facilitated the transition from vegetative growth to embryogenesis in *Arabidopsis* (Zuo et al., 2002). Gain-of-function of *WUS* mutant can trigger cell pluripotence and re-establishing new meristems in the cortex, which further reinforced the essential role of *WUS* in the initiation and maintenance of meristem *in planta* (Xu et al., 2005). Nevertheless, the underlying mechanisms of how somatic cell maintains plasticity and triggers cellular switch during the sex-free embryogenesis are still elusive, which is one of the top 25 profound as-yet-unanswered scientific questions (Vogel, 2005). During early somatic embryogenesis, while auxin signaling cascade induces the expression of *WUS* (Su et al., 2011), little is known about the underlying mechanisms for the RAM establishment.

The quiescent center (QC) is formed from apical descendant cell after asymmetric division of the hypophysis and essential in maintaining the identity of root stem cells (Möller and Weijers, 2009; Peris et al., 2010). WUSCHEL-RELATED HOMEOBOX 5 (WOX5) plays an analogous role in the QC of the RAM as WUS in organizing center (OC) of the SAM. PLETHORA1 (PLT1) and PLT2, two AP2-type transcription factors, are required to specify and maintain the stem–cell identity in the RAM (Galinha et al., 2007). In addition, transcription factors SCARECROW (SCR) and SHORTROOT (SHR) are necessary to maintain stem cell activity in the RAM (Sabatini et al., 2003). In this study, Dr Xian Sheng Zhang's lab exploited the marker lines of the above genes to investigate the specification and initiation of RAM during nongametic somatic embryogenesis. They detected almost simultaneously induced *WOX5* and *PLT2* expression in some regions of embryonic callus only 24 h after transferred onto 2,4-D free somatic embryo-inducing medium (SEIM), while the expression of *SCR* was detected later in 24–36 h. Further, the spatiotemporal expression patterns of *WUS* and *WOX5* were examined by tagged with distinct fluorescence protein markers. Nearly identical expression patterns of them were found after 24 h induction. The *WOX5* signals were then detected below the regions of *WUS* signals, suggesting that an early shoot-root axis had been established at this time. Su and colleagues also employed inducible antisense *WOX5* transgenic lines and *plt2* mutant to investigate their roles for embryonic root formation. Strikingly, significantly decreased number of somatic embryos (SEs) and severely defected SEs were produced from embryonic callus of *WOX5* antisense lines and *plt2-1* mutant respectively, confirmed that WOX5 and PLT2 are required for somatic embryogenesis, and further indicated the role of WOX5 in the RAM is comparable to that of WUS in the SAM.

In zygotic embryos, auxin and cytokinin display inverse correlation in specifying the root stem cell niche (Müller and Sheen, 2008). In this scenario, auxin signaling antagonizes cytokinin responses during zygotic embryogenesis by directly inducing the transcription of ARABIDOPSIS RESPONSE REGULATOR (ARR) 7 and ARR15, the two critical A-type ARR transcription factors to repress cytokinin signaling (Figure 1), which is essential in establishing the root stem cell niche (Müller and Sheen, 2008). Intriguingly, ARR7 and ARR15 expression is stimulated during root specification, but repressed by the auxin signal in the SAM of the embryo (Zhao et al., 2010). However, the interaction of auxin and cytokinin in the establishment of root stem cell niche in somatic embryogenesis is less clear. Su and her colleagues further traced auxin response signals by utilizing *DR5rev* promoter driven *VENUS* or *GFP* expression and found that its spatiotemporal expression patterns were overlapped with neither *WOX5:GFP* nor *PLT2:RFP*. Instead, they showed that *ARR7* and *ARR15*, which also can be induced by cytokinin (Werner and Schmülling, 2009), displayed overlapping expression patterns with *WOX5* rather than *WUS*. This remarkable evidence implied that cytokinin, but not auxin, is essential for initiation of the RAM of SE (Figure 1). This was further supported by the observation of defective SEs in the overexpressing lines of *ARR7* and *ARR15*, and double mutants of *ahk2ahk4* and *ahk3ahk4*. The authors finally demonstrated that cytokinin signaling strictly confined *WOX5* expression to the site of subsequently embryonic root meristem by *in situ* hybridization (Su et al., 2015).

**Figure 1:**
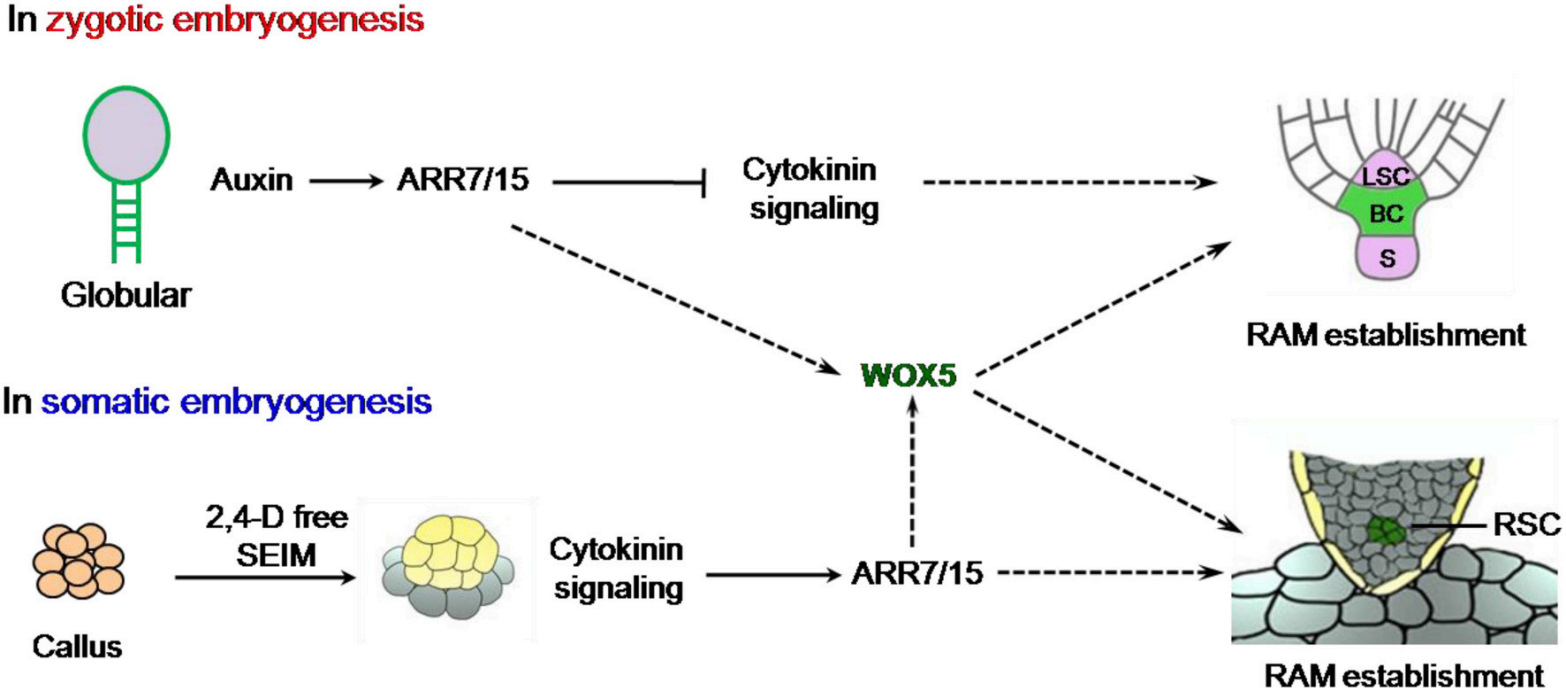
**Comparison of the roles of auxin and cytokinin during RAM specification in zygotic and somatic embryos**. The cells with green color represent established RAM niche in zygotic embryogenesis (ZE) and somatic embryogenesis (SE) respectively. The dashed lines stand for proposed molecular link but need to be further corroborated. S, Suspensor; LSC, Lens-Shaped Cell; BC, Basal Cell lineage; RSC, root stem cells of SE.

The establishment of the shoot-root axis is a crucial process for both zygotic and somatic embryogenesis. Despite the processes of the two kinds of embryogenesis are quite similar from globular to torpedo stage (Zimmerman, 1993), the underlying mechanisms seem to be different in at least two aspects. (1) The spatiotemporal expression patterns of *WUS* and *WOX* genes are distinct. In zygotic embryogenesis, *WUS* and *WOX5* are sequentially expressed with roughly 12 h interval. While during somatic embryogenesis they are simultaneously expressed in nearly overlapping regions of embryonic callus, indicating that SAM and RAM are almost initiated at the overlapping regional domains of callus and formed the apical-basal axis at early stages of somatic embryogenesis. (2) The roles of auxin gradient response and cytokinin signaling show discrepancy in zygotic and somatic embryogenesis (Figure 1). *In planta*, the proper establishment of auxin gradient response is essential for correct spatiotemporal cytokinin signaling, which causes the lower expression of *ARR7/ARR15* in the upper lens-shaped cell but higher expression in the lower large basal cell (Müller and Sheen, 2008). Cytokinin signaling plays a critical role during root stem cell niche formation in zygotic embryogenesis as chemical induced double mutant *arr7arr15* produced the defective RAM system. In here, Su and her colleagues' compelling results demonstrated that cytokinin signaling is also essential for RAM initiation in SE. Collectively, their results provide novel insights into the roles of cytokinin and auxin in the formation of shoot-root axis during somatic embryogenesis, and contribute to the ongoing investigation of the mechanisms of somatic embryogenesis. These knowledges are invaluable in propagating the endangered and indispensable plant species and in molecular breeding of crops through callus-based plant transformation in the near future.

## Author contributions

LW and KC conceived the research and wrote the manuscript.

### Conflict of interest statement

The authors declare that the research was conducted in the absence of any commercial or financial relationships that could be construed as a potential conflict of interest.
